# Verification That Mouse Chromosome 14 Is Responsible for Susceptibility to Streptozotocin in NSY Mice

**DOI:** 10.1155/2018/7654979

**Published:** 2018-11-21

**Authors:** Naru Babaya, Hironori Ueda, Shinsuke Noso, Yoshihisa Hiromine, Michiko Itoi-Babaya, Misato Kobayashi, Tomomi Fujisawa, Hiroshi Ikegami

**Affiliations:** ^1^Department of Endocrinology, Metabolism and Diabetes, Kindai University Faculty of Medicine, Osaka, Japan; ^2^Department of Molecular Endocrinology, Osaka University Graduate School of Medicine, Osaka, Japan; ^3^Health Care Center, Rinku General Medical Center, Osaka, Japan; ^4^Department of Applied Molecular Bioscience, Graduate School of Bioagricultural Sciences, Nagoya University, Aichi, Japan; ^5^Sakai City Medical Center, Osaka, Japan

## Abstract

**Introduction:**

Streptozotocin- (STZ-) induced diabetes is under polygenic control, and the genetic loci for STZ susceptibility are mapped to chromosome (Chr) 11 in Nagoya-Shibata-Yasuda (NSY) mice. In addition to Chr11, other genes on different chromosomes may contribute to STZ susceptibility in NSY mice. The aim of this study was to determine whether NSY-Chr14 contributes to STZ susceptibility and contains the STZ-susceptible region.

**Materials and Methods:**

A consomic C3H-14^NSY^ strain (R0: homozygous for NSY-derived whole Chr14 on the control C3H background), two congenic strains (R1: the region retained proximal and middle segments of NSY-Chr14 and R2: the region retained a proximal segment of NSY-Chr14), and parental NSY and C3H mice were intraperitoneally injected with a single injection of STZ at a dose of 175 mg/kg body weight at 12 weeks of age. Blood glucose levels and body weights were measured at days 0, 1, 2, 4, 5, 7, 8, and 14 after STZ injection. At day 14 after STZ injection, pancreata were dissected and fixed.

**Results:**

After STZ injection, blood glucose levels were significantly higher in R0 mice than in C3H mice. However, blood glucose levels in R0 mice were not as severely affected as those in NSY mice. In R1 and R2 mice, blood glucose levels were similar to those in C3H mice and were significantly lower than those in R0 mice. Body weights were decreased in NSY and R0 mice; however, this change was not observed in R1, R2, and C3H mice. Although islet tissues in all strains exhibited degeneration and cellular infiltration, histological changes in NSY and R0 mice were more severe than those in R1, R2, and C3H mice.

**Conclusions:**

These data demonstrated that NSY-Chr14 was a STZ-susceptible chromosome and that STZ susceptibility was mapped to the distal segment of NSY-Chr14.

## 1. Introduction

Streptozotocin (STZ) has been widely used to induce diabetes through pancreatic islet destruction in experimental animals [1–3]. STZ, a small molecule that resembles glucose, is taken up to bind glucose transporter-2 [1, 3]. In islet *β*-cells, STZ decomposes and damages DNA, which leads to cell death [1, 3]. Among inbred mouse strains, varying susceptibility to STZ-induced diabetes has been reported. Nonobese diabetic (NOD) mice, an inbred strain of type 1 diabetes [4], are extremely susceptible to *β*-cell destruction by STZ [5]. Although susceptibility to type 1 diabetes is primarily determined by the immunological factor, several studies have indicated that the intrinsic vulnerability of *β*-cells is also involved in susceptibility to type 1 diabetes, which suggests shared mechanisms between STZ-induced diabetes and type 1 diabetes [5–8].

Nagoya-Shibata-Yasuda (NSY) mice [9], an inbred strain of type 2 diabetes with moderate obesity and fatty liver, were established by selective breeding for glucose intolerance from an outbred colony, Jcl:ICR mice, from which NOD mice were also derived [10]. Both NSY and NOD mice are extremely STZ-sensitive strains [5, 7, 11], which suggests a shared genetic basis in the vulnerability of *β*-cells between these strains. *β*-cell fragility may be shared between type 1 and type 2 diabetes [6, 12, 13]. These lines of evidence indicate that three types of diabetes (STZ-induced, type 1, and type 2 diabetes) share common genetic factors and mechanisms. Therefore, the identification of STZ-susceptible genes is important to clarify the mechanism of *β*-cell vulnerability in type 1 and type 2 diabetes.

We previously identified three major quantitative trait loci (QTLs) for diabetes-related phenotypes (*Nidd1n*, *Nidd2n*, and *Nidd3n*) on chromosome (Chr) 11, 14, and 6, respectively [14]. A QTL for fatty liver (*Fl1n*) and a QTL for body weight (*Bw1n*) on Chr6 [15] were found using NSY and C3H (nondiabetic) mice. Subsequent studies using consomic C3H-11^NSY^ and C3H-14^NSY^ mice in which the entire NSY-Chr11 and NSY-Chr14 were introgressed onto the genetic background of control C3H mice clearly demonstrated that NSY-Chr11 and NSY-Chr14 harbor loci for diabetes [16]. Subsequent studies using consomic C3H-11^NSY^ mice indicated that NSY-derived Chr11 harbors susceptibility to STZ-induced diabetes [7, 11]. However, the STZ sensitivity of the C3H-11^NSY^ strain is not as strong as that of the NSY parental strain, which suggests that STZ-induced diabetes is under polygenic control and that genes on chromosomes other than Chr11 also contribute to STZ susceptibility [7, 11].

In this study, to detect novel loci related to STZ susceptibility, we focused on another diabetogenic chromosome, i.e., NSY-Chr14. C3H-14^NSY^ mice and their congenic strains were administered a single high dose of STZ, and the STZ sensitivities of these strains were compared with those of the parental strains.

## 2. Materials and Methods

### 2.1. Animals

We used five strains, namely, NSY [9, 10, 14, 17, 18], C3H/HeNcrj (C3H), consomic C3H-14^NSY^ (R0) [16], congenic R1, and R2 mice [19]. NSY mice were originally obtained from the Branch Hospital of Nagoya University School of Medicine. C3H mice were purchased from Charles River Laboratories (Kanagawa, Japan).

R0 mice, which were homozygous for the NSY-derived whole Chr14 on the control C3H background, were previously constructed [16] using the speed congenic method [20, 21]. Briefly, F1 male mice were obtained by mating (NSY × C3H). These males were mated with C3H females, and their progeny heterozygous for Chr14 were used for next generation. This backcross was repeated until all the markers for background typing became homozygous for C3H genotype, at which point the heterozygous consomic strains were obtained. The mice heterozygous for Chr14 were intercrossed to obtain mice homozygous for Chr14.

**Figure 1:**
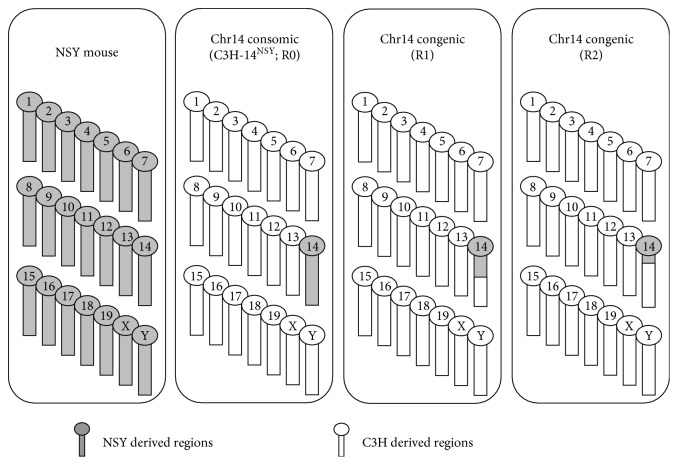
Schematic illustration of NSY, Chr14 of consomic mice (C3H-14^NSY^; R0) and congenic mice (R1 and R2), which carry NSY-derived susceptible regions onto a C3H-derived resistance background. Regions from NSY mice are shown in gray, and regions from C3H mice are shown in white.

**Figure 2:**
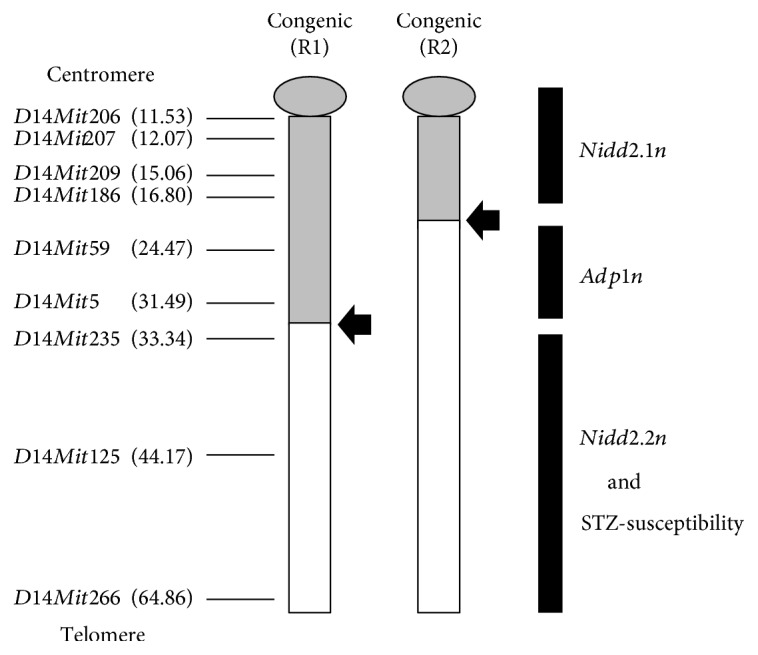
Schematic illustration of congenic mice. R1 possesses proximal and middle segments of NSY-Chr14 from the centromere to the recombinant position between *D14Mit5* and *D14Mit235*. R2 mice possess the proximal segment of NSY-Chr14 from the centromere to the recombinant position between *D14Mit186* and *D14Mit59*. Regions from NSY mice are shown in gray, and regions from C3H mice are shown in white. Arrows show each recombinant position. In parentheses, marker map positions from the centromere obtained from the Mouse Genome Database (http://www.informatics.jax.org). The diabetogenic loci (*Nidd2.1n* and *Nidd2.2n*) and the adiposity locus (*Adp1n*), which were reported in our previous study [19], and STZ-susceptibility locus, which was demonstrated in this study, are shown in black bars.

Recently, we also constructed two novel congenic lines, R1 and R2, obtained from R0 (Figure 1) [19]. Briefly, heterozygous R1 and R2 male mice were produced by mating (R0 × C3H) F1 with C3H and selecting males that possessed the genomic region of interest on Chr14, and then, the heterozygous R1 and R2 male mice were mated with C3H females. Their progeny with the genomic region of interest were intercrossed to obtain homozygous mice. R1 mice possess the proximal and middle segments of NSY-Chr14 from the centromere to the recombinant position between *D14Mit5* and *D14Mit235* (Figure 2). R2 mice possess the proximal segment of NSY-Chr14 from the centromere to the recombinant position between *D14Mit186* and *D14Mit59* (Figure 2). The positions of the diabetogenic loci (*Nidd2.1n* and *Nidd2.2n*) and the adiposity locus (*Adp1n*), which were reported in our previous study [19], are shown in Figure 2.

These mice were maintained by brother-sister mating and under specific pathogen-free conditions in the animal facilities of Osaka University Graduate School of Medicine. All mice had free access to tap water and a standard diet (CRF-1: Oriental Yeast, Tokyo, Japan) in a temperature-controlled room (22–25°C) on a 12 h light-dark cycle (6:00–18:00 h). The animal protocols used for this study were approved by the Osaka University Graduate School of Medicine Committee on Animal Welfare. Male mice were used for all experiments.

### 2.2. Protocols

NSY, C3H, R0, R1, and R2 mice received a single injection of STZ at a dose of 175 mg/kg body weight at 12 weeks of age. STZ was dissolved in sodium citrate buffer (Wako Pure Chemical Industries, Ltd., Osaka, JAPAN) and immediately injected intraperitoneally. Blood glucose levels and body weights were measured ad lib at days 0, 1, 2, 4, 5, 7, 8, and 14 after STZ injection. Blood glucose levels were determined by the glucose oxidase method using Glutest Ace (Sanwa Kagaku Kenkyusho Co., Ltd., Nagoya, Japan) in which the detection limit was 33.34 mmol/l. In this study, glucose values greater than 33.34 mmol/l were reported as 33.34 mmol/l. Mice with glycemia greater than 16.7 mmol/l were considered hyperglycemic because the NSY strain is a model of type 2 diabetes; therefore, the ad lib blood glucose level can exceed 11.1 mmol/l. Some of the data from NSY and C3H mice have been previously reported [7, 11] and were reanalyzed in this study.

### 2.3. Histological Examination

STZ-treated mice were killed under sevoflurane anesthesia at day 14 after injection, and pancreata were dissected and fixed in neutralized 10% formalin. Paraffin sections of those tissues were stained with hematoxylin-eosin by the standard method. The cellular infiltration in and around islets in NSY, C3H, R0, R1, and R2 mice was graded (0: normal islet; 1: peri-insulitis or <25% of *β*-cell area infiltrated; and 2: more than 25% of *β*-cell area infiltrated). All islets were evaluated by an observer, blinded with respect to the origin of the sections.

### 2.4. Statistical Analysis

All values are expressed as the mean ± SEM. Statistical analysis was performed by the Mann–Whitney *U* test or one-way analysis of variance (ANOVA) with post hoc tests (Dunnett's multiple comparison tests). Survival curves were analyzed with the log-rank test. Statistical tests were performed using the Prism software (GraphPad Prism®). *P* < 0.05 was considered statistically significant.

## 3. Results

### 3.1. STZ Sensitivity in Consomic C3H-14^NSY^; R0 (Figure 3, Supplemental Figure 1)

**Figure 3:**
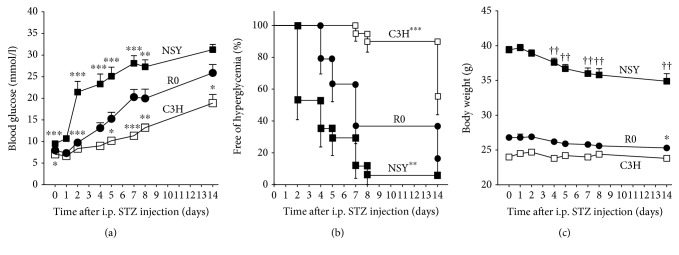
STZ sensitivity at 12 weeks of age in NSY (*n* = 17; black squares), R0 (*n* = 19; black circles), and C3H mice (*n* = 20; white squares). Glucose concentrations and body weight are measured ad lib at days 0, 1, 2, 4, 5, 7, 8, and 14 days after STZ injection. Data are expressed as the mean ± SEM. (a) Blood glucose concentrations. Four NSY mice were dead (one at day 3 and three at days 9–13). Three R0 mice were dead at days 9–13. Because these dead mice showed hyperglycemia before death, the glucose levels after death were reported as 33.34 mmol/l, which is the detection limit of the glucose sensor. ^∗^ *p* < 0.05, ^∗∗^ *p* < 0.01, ^∗∗∗^ *p* < 0.001 compared with R0 (one-way ANOVA with post hoc test (Dunnett's multiple comparison tests)). (b) The percentage of animals free of hyperglycemia. Mice with glycemia higher than 16.7 mmol/l were considered hyperglycemic. ^∗∗^ *p* < 0.01, ^∗∗∗^ *p* < 0.001 compared with R0 (log-rank test). (c) Body weight changes. Four NSY and three R0 mice were dead (described previously). This figure does not contain the body weight data after death. ^∗^ *p* < 0.05 compared with the basal body weight of R0 and ^††^ *p* < 0.01 compared with the basal body weight of NSY (Mann–Whitney *U* test).

Three R0 mice were dead at days 9–13 due to hyperglycemia. R0 mice exhibited significantly higher blood glucose levels after STZ injection than did C3H mice (Figure 3(a)). Life table analysis demonstrated a significant difference in the survival curves of mice free from hyperglycemia between R0 and C3H mice (Figure 3(b), *p* < 0.001). At 14 days post-STZ injection, body weights (Figure 3(c)) were significantly reduced in R0 mice (−5.6% from the basal, *p* < 0.05). In contrast, no body weight reduction was observed in C3H mice (+0.8% from the basal, *p* = 0.35). These results indicate that introgression of a single Chr14 from STZ-sensitive NSY mice converted STZ-resistant C3H mice to STZ-sensitive mice.

However, blood glucose levels in R0 mice were not as severe as those in NSY mice (Figure 3(a)). Four NSY mice were dead (one at day 3 and three at days 9–13) because of hyperglycemia. Compared with R0 mice, NSY mice exhibited significantly higher blood glucose levels after STZ injection, and life table analysis demonstrated a significant difference between the survival curves (Figure 3(b), *p* < 0.01). At 14 days after STZ injection, the reduction in the body weights of NSY mice (−11.4% from the basal, *p* < 0.01) was more severe than that of R0 mice (Figure 3(c)). These results indicate that the sensitivity of R0 mice to STZ is not as strong as that of NSY mice, which suggests the contribution of other chromosomes in addition to Chr14 to STZ susceptibility.

### 3.2. STZ Sensitivity in Congenic Mice; R1 and R2 (Figure 4, Supplemental Figure 1)

**Figure 4:**
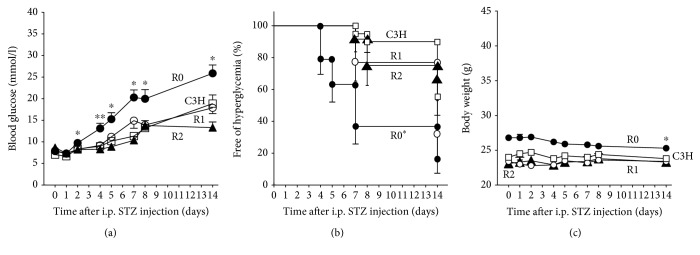
STZ sensitivity at 12 weeks of age in R0 (*n* = 19; black circles), C3H (*n* = 20; white squares), R1 (*n* = 13; white circles), and R2 mice (*n* = 12; black triangles). Glucose concentrations and body weight are measured ad lib at days 0, 1, 2, 4, 5, 7, 8, and 14 days after STZ injection. Data are expressed as the mean ± SEM. (a) Blood glucose concentrations. The values of glucose in the R0 were reported as shown in Figure 3 legend. ^∗^ *p* < 0.05, ^∗∗^ *p* < 0.01 compared with R1 (one-way ANOVA with post hoc test (Dunnett's multiple comparison tests)). (b) The percentage of animals free of hyperglycemia. Mice with glycemia higher than 16.7 mmol/l were considered hyperglycemic. ^∗^ *p* < 0.05 compared with R1 (log-rank test). (c) Body weight changes. The values of body weight in R0 were reported as shown in Figure 3 legend. ^∗^ *p* < 0.05 compared with the basal body weight of R0 (Mann–Whitney *U* test).

Glucose levels after STZ injection in R1 mice were similar to those in C3H mice and were significantly lower than those in R0 mice (Figure 4(a)). The cumulative incidence of STZ-induced hyperglycemia in R1 mice was similar to that in C3H mice and was significantly lower than that in R0 mice (Figure 4(b), *p* < 0.05). In R1 mice, no reduction in body weight was observed (+0.0% from the basal, *p* = 0.94).

Glucose levels after STZ injection in R2 mice were similar to those in R1 and C3H mice (Figure 4(a)). Life table analysis demonstrated no difference in the survival curves of mice free from hyperglycemia among congenic (R1 and R2) and C3H mice (Figure 4(b)). In R2 mice, no reduction in body weight was observed (+0.9% from the basal, *p* = 0.82).

Since R0 mice but not R1 and R2 mice showed STZ sensitivity, the data in the present study indicate that the distal region of Chr14 retained in R0 mice but not in R1 and R2 mice plays an important role in STZ susceptibility (Figure 2).

### 3.3. Histological Phenotype in NSY, C3H, R0, R1, and R2 Mice (Figure 5, Supplemental Figure 2)

In all strains, the histological sections of islet tissues at day 14 showed degeneration. Architectural disarray of pancreatic islets and cellular infiltration were observed. Histological changes in NSY and R0 mice were more severe than those in R1, R2, and C3H mice. Apparent signs of cellular infiltration in or around the pancreatic islets were also observed in all strains. Cellular infiltration in NSY and R0 mice was more severe than that in R1, R2, and C3H mice (Supplemental Figure 2).

## 4. Discussion

We previously reported that Chr11 harbors susceptibility to STZ-induced diabetes in NSY mice [7, 11]. However, Chr11 was insufficient to explain all STZ sensitivity in NSY mice. In this study, we identified an additional STZ-susceptible chromosome, Chr14, and mapped STZ susceptibility to the distal region of Chr14 (from *D14Mit5* to the telomere).

In our previous study, we established two congenic strains in which limited segments of NSY-Chr14 were introgressed onto control C3H background genes [19]. One congenic strain, termed R1, possessed a proximal half segment of NSY-Chr14 (<33.34 cM from the centromere to *D14Mit235*), and the other congenic, termed R2, possessed a more limited segment of NSY-Chr14 (<24.47 cM from the centromere to *D4Mit59*). Analysis of these congenic strains demonstrated that a locus termed *Nidd2.2n* in the distal segment of NSY-Chr14 affects fasting glucose, postchallenge hyperglycemia, and insulin resistance [19]. A locus for STZ susceptibility localized to the distal region of NSY-Chr14 in the present study overlaps with the region for *Nidd2.2n*. Further studies with congenic strains with the distal region of NSY-Chr14 are necessary for fine mapping and identification of the responsible gene.

The distal region of Chr14 where STZ susceptibility was mapped in the present study was not previously linked to STZ-induced diabetes. Gonzalez et al. reported that two genetic loci for STZ susceptibility were identified on Chr 9 and Chr 11 in NOD mice and suggested that the two loci were insufficient to predict resistance or sensitivity to STZ-induced diabetes [5]. In our previous and present studies, the STZ sensitivity of consomic C3H-11^NSY^ and C3H-14^NSY^ mice was less than that of NSY mice, suggesting that STZ-induced diabetes is under polygenic control. We do not know whether NSY-Chr11 and NSY-Chr14 are sufficient for the full expression of the STZ sensitivity of NSY mice. Analysis of C3H-11^NSY^14^NSY^ mice [16] containing both NSY-Chr11 and NSY-Chr14 on the C3H background will lead to the answer.

A single high dose of STZ is used for experiments attempting to cause type 1 diabetes by direct toxicity, and glucose homeostasis deterioration rapidly arises within a few days [22]. Although only 10% of STZ-treated mice that received a single high dose exhibited mononuclear cell infiltration in pancreatic islets, more than 60% of STZ-treated mice that were treated with a single low dose exhibited this phenomenon [23]. In this study, we used a single high dose of STZ in NSY mice. Hyperglycemia began to appear within 48 h after STZ injection. Mononuclear cell infiltration was detected in all examined pancreata. However, we do not know why NSY mice had severe mononuclear cell infiltration despite the single high-dose STZ injection. One possible reason is that pancreata were dissected at 14 days after STZ injection in our study, whereas dissection occurred within 1 week after the development of hyperglycemia in a previous study [23]. Another possible reason is simply strain differences.

## 5. Conclusion

The present study demonstrated that NSY-Chr14 was a STZ-susceptible chromosome and that the STZ-susceptible region was located in the distal segment of NSY-Chr14. Construction of new congenic strains will lead to fine mapping and identification of causal variants of the genes responsible for STZ susceptibility in the NSY mouse.

## Figures and Tables

**Figure 5:**
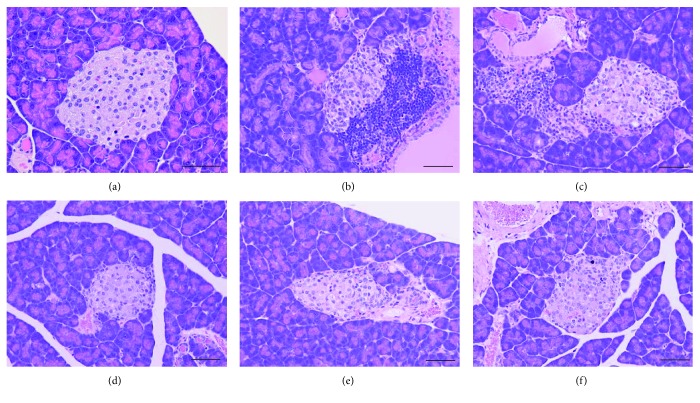
Pancreatic islets stained with hematoxylin-eosin. Scale bars, 50 *μ*m. (a) An intact islet from non-STZ-injected NSY mice at 24 weeks of age. Inflammatory changes were not observed. (b–f) Typical islets from STZ-injected NSY (b), R0 (c), R1 (d), R2 (e), and C3H (f) mice at day 14 after STZ injection.

## Data Availability

The data used to support the findings of this study are included within the article.

## Supplementary Materials

Supplementary Materials Supplemental Figure 1: blood glucose concentrations in NSY, C3H, R0, R1, and R2 mice ad lib at days 0, 1, 2, 4, 5, 7, 8, and 14 after STZ injection. Four NSY mice and three R0 mice died during follow-up. The values of glucose in the dead NSY and R0 mice were reported as shown in Figure 3 legend. ^∗∗^ *p* < 0.01, ^∗∗∗^ *p* < 0.001 (higher) and ^†^ *p* < 0.05, ^††^ *p* < 0.01, ^†††^ *p* < 0.001 (lower) compared with R0 (one-way ANOVA with post hoc test (Dunnett's multiple comparison tests)). Supplemental Figure 2: degree of cellular infiltration in and around islets in NSY, C3H, R0, R1, and R2 mice. The cellular infiltration was graded (normal islet: white; peri-insulitis or <25% 346 of *β*-cell area infiltrated: gray; more than 25% of *β*-cell area infiltrated: black). Statistical analysis was performed by the Mann–Whitney *U* test. n.s.: not significant.
