# Supplementary figures and images for: Verification That Mouse Chromosome 14 Is Responsible for Susceptibility to Streptozotocin in NSY Mice
Source: Int J Endocrinol. 2018 Nov 21;2018:7654979. doi: 10.1155/2018/7654979 (PMC6280298; doi:10.1155/2018/7654979)

**Supplemental Figure 1**

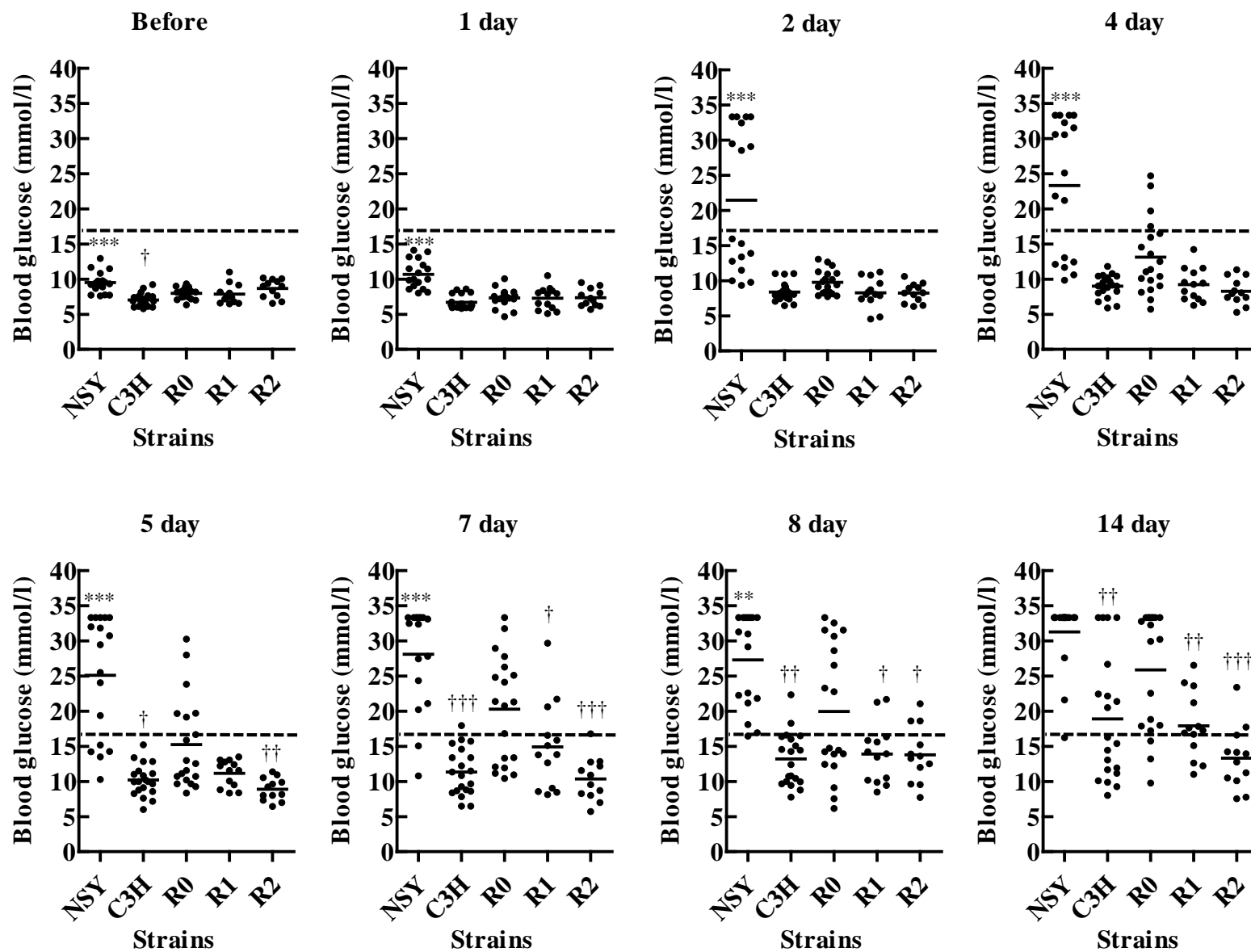

## Supplemental Figure 2

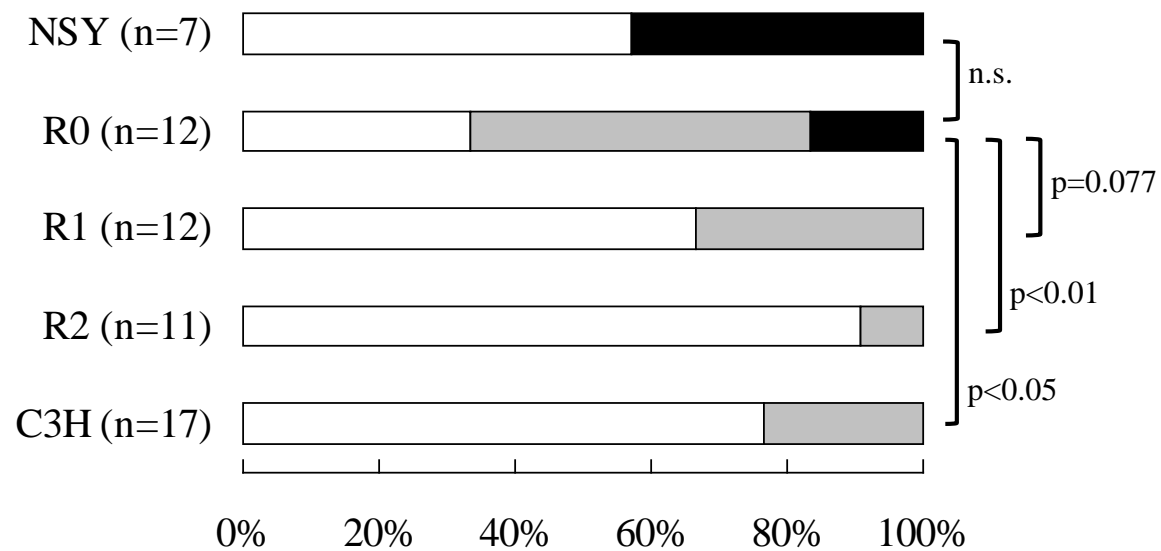

Supplement: Supplementary Materials — Supplemental Figure 1: blood glucose concentrations in NSY, C3H, R0, R1, and R2 mice ad lib at days 0, 1, 2, 4, 5, 7, 8, and 14 after STZ injection. Four NSY mice and three R0 mice died during follow-up. The values of glucose in the dead NSY and R0 mice were reported as shown in Figure 3 legend. ∗∗ p < 0.01, ∗∗∗ p < 0.001 (higher) and † p < 0.05, †† p < 0.01, ††† p < 0.001 (lower) compared with R0 (one-way ANOVA with post hoc test (Dunnett's multiple comparison tests)). Supplemental Figure 2: degree of cellular infiltration in and around islets in NSY, C3H, R0, R1, and R2 mice. The cellular infiltration was graded (normal islet: white; peri-insulitis or <25% 346 of β-cell area infiltrated: gray; more than 25% of β-cell area infiltrated: black). Statistical analysis was performed by the Mann–Whitney U test. n.s.: not significant. [file 7654979.f1.pdf]
